# Interspecies interactions mediated by arginine metabolism enhance the stress tolerance of *Fusobacterium nucleatum* against *Bifidobacterium animalis*

**DOI:** 10.1128/spectrum.02235-24

**Published:** 2025-01-27

**Authors:** Zhongkun Zhou, Mengyue Yang, Hong Fang, Yuqing Niu, Juan Lu, Yunhao Ma, Baizhuo Zhang, Hongmei Zhu, Peng Chen

**Affiliations:** 1 School of Pharmacy, Lanzhou University, Lanzhou, China; University of the Pacific, San Francisco, California, USA

**Keywords:** colorectal cancer, *Bifidobacterium animalis*, *Fusobacterium nucleatum*, whole-genome sequencing, metabolic network reconstruction

## Abstract

Colorectal cancer (CRC) is a common cancer accompanied by microbiome dysbiosis. Exploration of probiotics against oncogenic microorganisms is promising for CRC treatment. Here, differential microorganisms between CRC and healthy control were analyzed. Antibacterial experiments, whole-genome sequencing, and metabolic network reconstruction were combined to reveal the anti-*Fusobacterium nucleatum* mechanism, which was verified by co-culture assay and mendelian randomization analysis. Sequencing results showed that *F. nucleatum* was enriched in CRC, yet *Bifidobacterium animalis* decreased gradually from healthy to CRC. Additionally, *F. nucleatum* could be inhibited by *B. animalis*. Whole-genome sequencing of *B. animalis* showed high phylogenetic similarity with known probiotic strains and highlighted its functions for amino acid and carbohydrate metabolism. Metabolic network reconstruction demonstrated that cross-feeding and specific metabolites (acidic molecules, arginine) had a great influence on the coexistence relationship. Finally, the arginine supplement enhanced the competitive ability of *F. nucleatum* against *B. animalis*, and the mendelian randomization and metagenomic sequencing analysis confirmed the positive relationship among *F. nucleatum*, arginine metabolism, and CRC. Thus, whole-genome sequencing and metabolic network reconstruction are valuable for probiotic mining and patient dietary guidance.

**IMPORTANCE:**

Using probiotics to inhibit oncogenic microorganisms (*Fusobacterium nucleatum*) is promising for colorectal cancer (CRC) treatment. In this study, whole-genome sequencing and metabolic network reconstruction were combined to reveal the anti-*F. nucleatum* mechanism of *Bifidobacterium animalis*, which was verified by co-culture assay and mendelian randomization analysis. The result indicated that the arginine supplement enhanced the competitive ability of *F. nucleatum*, which may be harmful to *F. nucleatum*-infected CRC patients. *B. animalis* is a potential probiotic to relieve this dilemma. Thus, using in silico simulation methods based on flux balance analysis, such as genome-scale metabolic reconstruction, provides valuable insights for probiotic mining and dietary guidance for cancer patients.

## INTRODUCTION

Colorectal cancer (CRC) is one of the most common cancers in clinics, with incidence ranking third and mortality ranking second globally in both sexes, and it accounts for nearly 10% of cancer deaths (1). CRC is one multifactorial disease. Besides inheritance, environmental factors make a great contribution. Especially, the human microbiome, including the bacteria, archaea, eukaryotes, viruses, their genomes, and the surrounding environmental conditions, plays a key role in human health and is proposed as a new hallmark of cancer (2, 3). The advance of multi-omics and bioinformatics greatly deepens our understanding of their roles.

In the development of CRC, microbiota dysbiosis is accompanied by the enrichment of harmful bacteria and depletion of beneficial microbes, which are also reflected by the metabolism disorder (4). Several pathogenic bacteria have been proven to participate in the activation, progression, metastasis, and drug resistance processes of CRC, such as *Fusobacterium nucleatum*, pks+ *Escherichia* coli, BFT-producing *Bacteroides fragilis*, *Peptostreptococcus anaerobius*, *Parvimonas micra*, *Peptostreptococcus stomatis,* etc. (5). *F. nucleatum* is a common oral pathogenic bacterium and was found to be prevalent in CRC patients (6). Further studies demonstrate that it can bind to tumor-expressed Gal-GalNAc in a Fap2-dependent manner (7), and increase tumor multiplicity by recruiting tumor-infiltrating myeloid cells and inducing IL-8 secretion (8, 9). FadA, another toxin secreted by *F. nucleatum*, binds to E-cadherin and activates β-catenin signaling, thus promoting colorectal carcinogenesis (10). Additionally, through exosomes and m6A modification regulation, it contributes to colorectal cancer metastasis. *F. nucleatum* can promote colorectal cancer resistance to chemotherapy by activating the autophagy pathway through the TLR4-MYD88 pathway and upregulating BIRC3 expression (11, 12). Therefore, eliminating *F. nucleatum* will be beneficial for CRC treatment.

It was found that metronidazole could reduce *Fusobacterium* load, cancer cell proliferation, and overall tumor growth (13), yet broad-spectrum antibiotics will induce gut microbiota disorder and the emergence of drug-resistant bacteria. Therefore, several strategies were developed to resolve this dilemma. Recently, a minimalistic, biomimetic nano vaccine through integrating immunostimulatory adjuvant cholesterol-modified CpG oligonucleotides into the autologously derived *F. nucleatum* membranes successfully enhanced chemotherapy efficacy and reduced cancer metastasis (14). Another study constructed a phage-guided biotic–abiotic hybrid nanosystem, which augmented the efficiency of chemotherapy treatments of *F. nucleatum*-related CRC (15). Other targeted treatment strategies include nanoliposome design, natural product screening, and drug rediscovery (16–18). Besides the methods above, using probiotics to combat *F. nucleatum* is a promising direction. It was reported that a bacteriocin-producing *Streptococcus salivarius*, *Saccharomyces cerevisiae* JKSP39, and *Akkermansia muciniphila* could alleviate the inflammation induced by *F. nucleatum* without destruction of the gut microbiome (19–21). Compared with narrow-spectrum antibiotic development, probiotic therapy is safer, can generate broader regulation, and lasts for a longer period by colonization. Motivated by the success of treating *Clostridium difficile* infection with SER-109, discovering new probiotics to treat *F. nucleatum*-infected CRC is tempting (22). However, elucidating the antibacterial mechanism before clinical application is essential.

Using the methods of molecular biology to reveal specific mechanisms is necessary, yet the process may be time-consuming and full of difficulties. Integration of omics and bioinformatics can shed light on the discovery. For example, mining antimicrobial peptides from the human microbiome with a machine learning-based approach greatly accelerated the discovery process (23, 24). Furthermore, the ecology and evolution of microbiota is complex and dynamic. Competition, cooperation, commensalism, etc. indicate coexistence network and metabolic interaction among different microbes (25). Using *in silico* simulation methods based on flux balance analysis, such as genome-scale metabolic reconstruction, provides valuable guidance for the study of host-microbes and microbe-microbe interactions (26).

In this study, we found that *F. nucleatum* was prevalent in CRC patients globally, but *Bifidobacterium animalis* was depleted. *B. animalis* can inhibit *F. nucleatum* in exploitation manner. To reveal potential mechanisms, whole-genome sequencing and metabolic network reconstruction were performed, which emphasized the importance of metabolic interactions.

## RESULTS

### *F. nucleatum* is prevalent in CRC patients globally while *B. animalis* is depleted

**Fig 1:**
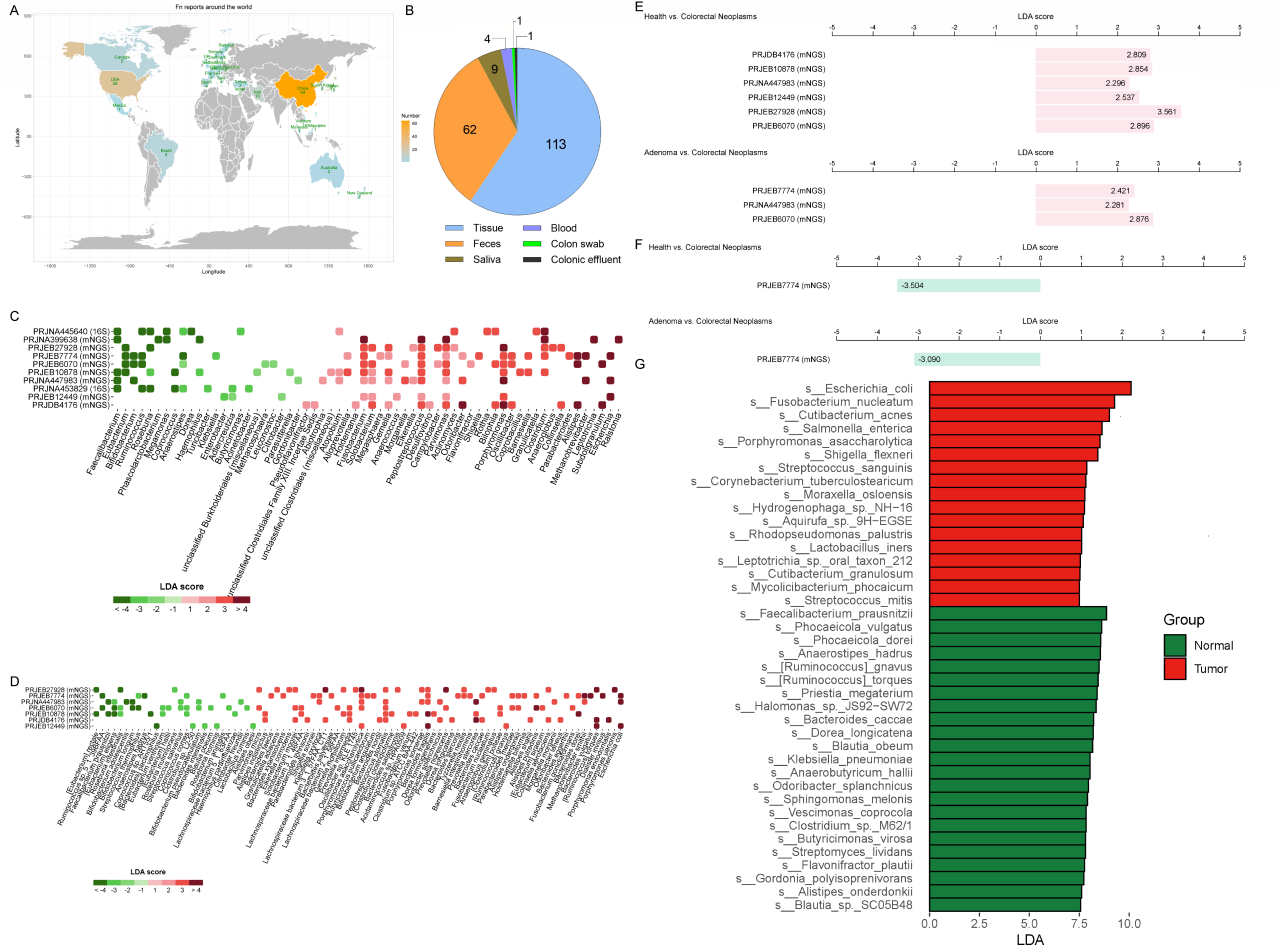
Abundance changes of CRC-related microbes. Global distribution of CRC-related *F. nucleatum* studies (**A**). Sample types of *F. nucleatum* studies (**B**). Differential genera (**C**) and species (**D**) between CRC and healthy control based on Linear discriminant analysis Effect Size analysis. The abundance changes of *F. nucleatum* between health and colorectal neoplasms, and between adenoma and colorectal neoplasms (**E**). The abundance changes of *B. animalis* between health and colorectal neoplasms, and between adenoma and colorectal neoplasms (**F**). The differential microorganisms of intra-tumoral microbiota (analyzed using TCMbio database) (**G**).

*F. nucleatum*, as one opportunistic pathogen, is reported to be related to many diseases, such as CRC, breast cancer, esophageal squamous cell carcinoma, atherosclerosis, and periodontitis (27). In this study, we focused on CRC and collected studies related to *F. nucleatum*, which included high throughput sequencing and quantitative PCR (qPCR) methods. Studies without healthy control were excluded. Finally, 174 studies were analyzed, containing 28 countries. China, USA, and Japan were the top three countries (Fig. 1A). Among these reports, 113 studies used tissue samples, 62 studies used feces, and other sample types including saliva, blood, colon swab, and colonic effluent (Fig. 1B). For CRC diagnosis, feces, and saliva were the most common samples for their non-invasive and convenient advantages (4). Next, the differential microorganisms between CRC and healthy control were analyzed using the GMrepo database (28). In multiple high throughput sequencing cohorts (16S rRNA and shotgun sequencing), *Faecalibacterium*, *Eubacterium*, and *Bifidobacterium* were the dominant genera depleted in CRC patients, yet *Fusobacterium*, *Peptostreptococcus*, and *Porphyromonas* were the main microbes enriched in CRC patients (Fig. 1C). At the species level, corresponding representative microorganisms were *Eubacterium rectale*, *Faecalibacterium prausnitzii*, *Bifidobacterium adolescentis*, *Bifidobacterium animalis*, *Porphyromonas asaccharolytica*, *Peptostreptococcus stomatis*, and *Fusobacterium nucleatum* (Fig. 1D). In addition, from healthy to adenoma and colorectal neoplasms, the abundance of *F. nucleatum* gradually increased while *B. animalis* decreased (Fig. 1E and F). Additionally, the enrichment of *F. nucleatum* was verified by the intra-tumoral microbiome compared with that in healthy tissue (Fig. 1G). Therefore, *F. nucleatum* is prevalent in CRC patients but *B. animalis* is reduced along the progression of CRC.

### *B. animalis* inhibits *F. nucleatum* in exploitation manner

**Fig 2:**
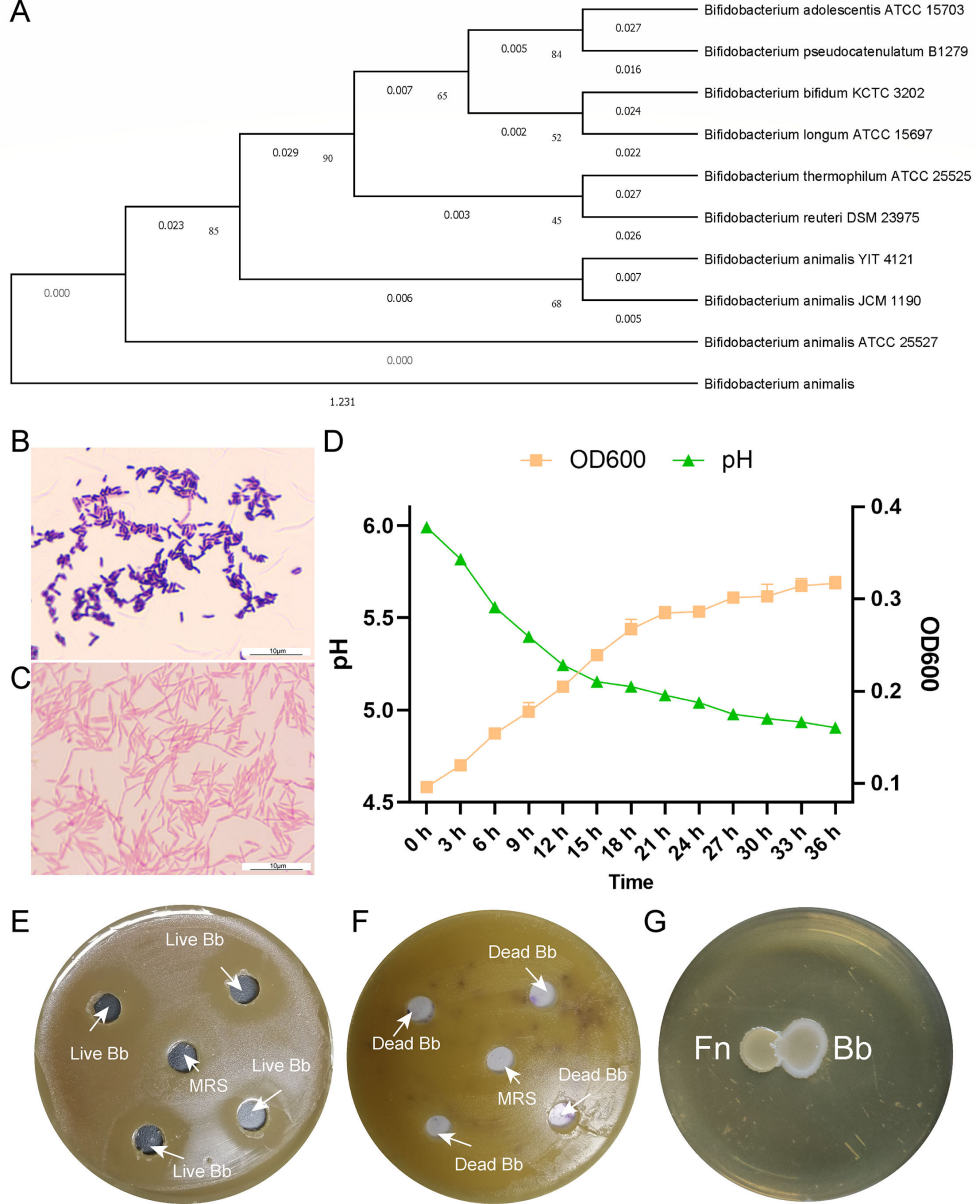
*B. animalis* inhibits *F. nucleatum*. Phylogenetic tree of *B. animalis* based on 16S rRNA gene sequencing (**A**). Gram’s stain of *B. animalis* (**B**) and *F. nucleatum* (**C**). Growth curve and pH changes of *B. animalis* cultured in MRS medium (**D**). Inhibition zone of *B. animalis* against *F. nucleatum* on GAM plate using MRS as control (*F. nucleatum* was spread on the plate and cultured for 24 h, then 6 mm wells were built. The well in the middle of the plate contained MRS medium as control, and the other wells contained live Bb.) (**E**). Inhibition zone of heat-killed *B. animalis* against *F. nucleatum* on GAM plate (**F**). Co-cultivation of *B. animalis* and *F. nucleatum* on GAM plate (**G**).

Considering the overgrowth of harmful bacteria and the decrease of beneficial microbes, restoring gut microecology to inhibit *F. nucleatum* through supplementing probiotics will be effective. Then we tested the antibacterial activity of *B. animalis*. It was used for the following reasons: first, according to our previous study based on 16S rRNA sequencing, *Bifidobacterium* is decreased in CRC patients compared with healthy people, and it ranks first in the importance contribution list of random forest model for CRC diagnosis (17). Second, the metagenomic sequencing studies demonstrated that *B. animalis* is depleted in CRC patients (Fig. 1D) (29). Third, *B. animalis* tolerates acid and oxidative stress. Fourth, it exists in the large intestines of most mammals, including humans. Fifth, it is safe (30). The *B. animalis* used in this study was isolated from healthy infant feces. First, the 16S rRNA sequencing confirmed that it belonged to *Bifidobacterium animalis* subsp. *lactis* strain with sequence similarity exceeding 99% (Fig. 2A). Gram’s stain showed that it was a rod-like Gram-positive bacterium (Fig. 2B), and *F. nucleatum* was shuttle shaped Gram-negative bacterium (Fig. 2C). Next, the growth curve of *B. animalis* showed that it reached the platform period at the 21st hour. Meanwhile, the pH of the culture medium decreased gradually and finally reached 5.15 (Fig. 2D). When *B. animalis* was inoculated to the plate containing *F. nucleatum*, the circle around the well indicated that *F. nucleatum* growth was inhibited, while de man, rogosa, and sharpe (MRS) medium could not inhibit *F. nucleatum* (Fig. 2E). Furthermore, when heat-killed *B. animalis* was added, the inhibition zone disappeared (Fig. 2F), indicating that live *B. animalis* played a key role. When *B. animalis* and *F. nucleatum* were inoculated on the Gifu Anaerobic Medium (GAM) plate at the same time, it showed that *B. animalis* had an advantage in growth competition and the relationship between them was classified as exploitation (+/-) (Fig. 2G). Therefore, *B. animalis* is a potential probiotic to treat *F. nucleatum*-infected CRC.

### Whole-genome sequencing reveals potential anti-*F*. *nucleatum* mechanisms

**Fig 3:**
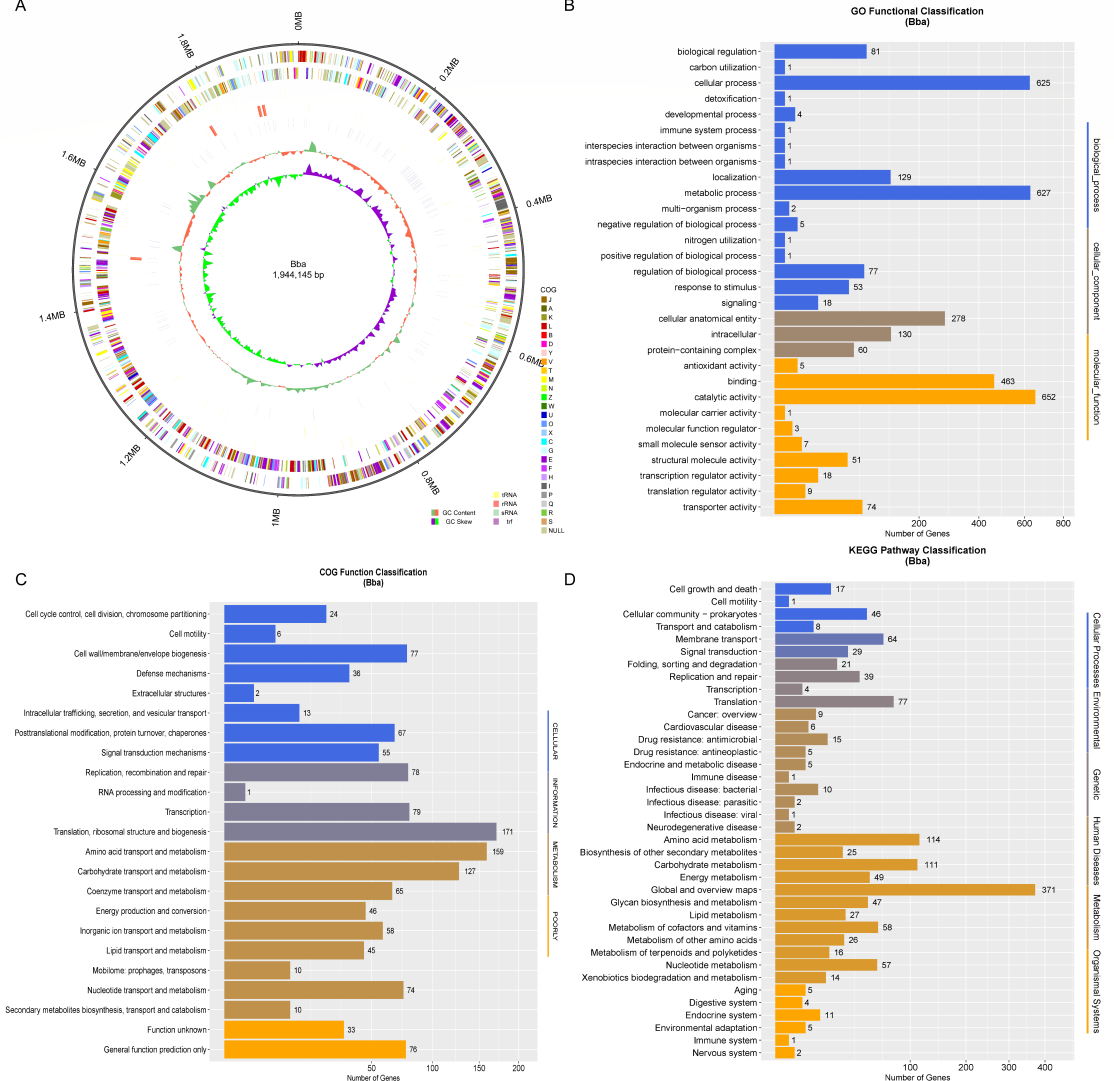
Whole-genome sequencing and function annotations of *B. animalis*. Genome circular of *B. animalis* (from the inner layer to the outer layer, represents genome size, forward strand gene, colored according to a cluster of orthologous groups [COG] classification, reverse strand gene, forward strand ncRNA, reverse strand ncRNA, repeat sequences, GC content, and GC-SKEW, respectively) (**A**). Gene Ontology (GO) (**B**), COG (**C**), and Kyoto Encyclopedia of Genes and Genomes (KEGG) (**D**) functional classifications of *B. animalis* genes.

To get a deeper understanding of the probiotic potential and anti-*F*. *nucleatum* mechanisms, whole-genome sequencing was performed. A 1,283 Mb (659×) BGISEQ Data and 8,164 Mb PacBio Data (4,199×) were obtained. The genome size of *B. animalis* is 1,944,145 bp, containing 1,617 genes, 53 tRNA, 468 5s rRNA, 6,104 16s rRNA, 12,351 23s rRNA, and 125 small RNA (Table S1; Fig. 3A). Furthermore, 122 tandem repeat (84 minisatellite DNA and 16 microsatellite DNA) were identified. Bacteria with prophage are called lysogenic bacteria. The presence of prophage sequences may also allow some bacteria to antibiotic resistance, adapt to the environment, improve adhesion, or bacteria pathogenicity. No prophage was identified in the *B. animalis* genome. Clustered regularly interspaced short palindromic repeats (CRISPR) sequences play a key role in a bacterial defense system, and six CRISPR were obtained with the length ranging from 83 to 1,404 bp.

Next, several databases were used for *B. animalis* genome function annotation. A total of 68, 2, 102, 1,352, 641, 1,161, 2, 1,030, 1,060, 1,616, and 349 genes were annotated in VFDB, ARDB, CAZY, IPR, SWISS-PROT, COG, CARD, GO, KEGG, NR, and T3SS, respectively (Table S2). For Gene Ontology (GO) functional classification, catalytic activity, metabolic process, cellular process, binding, and cellular anatomical entity were the top five functions, highlighting its catalytic and metabolic capacity (Fig. 3B). For cluster of orthologous group (COG) functional classification, most genes belonged to translation, ribosomal structure and biogenesis, amino acid transport and metabolism, and carbohydrate transport, and metabolism functions (Fig. 3C). In terms of Kyoto Encyclopedia of Genes and Genomes (KEGG) pathway classification, global and overview maps, amino acid metabolism and carbohydrate metabolism were the top three pathways (Fig. 3D). *F. nucleatum* is intolerant to acid (31). According to the KEGG annotations, we speculated that acidic metabolites produced by *B. animalis* during amino acid metabolism and carbohydrate metabolism contributed to anti-*F*. *nucleatum* activity. It was acknowledged that short-chain fatty acids (SCFA) are the major end products of carbohydrate metabolism in bifidobacteria (32). Additionally, in the preliminary conversion process of amino acid metabolism, α-Keto acid is the main acidic metabolite (33). Therefore, the acidic small molecules during the amino acid and carbohydrate metabolism, such as short-chain fatty acid or α-Keto acid, may contribute to the anti-*F*. *nucleatum* activity.

### Comparative analysis of specific functions from different strains of *Bifidobacterium*

**Fig 4:**
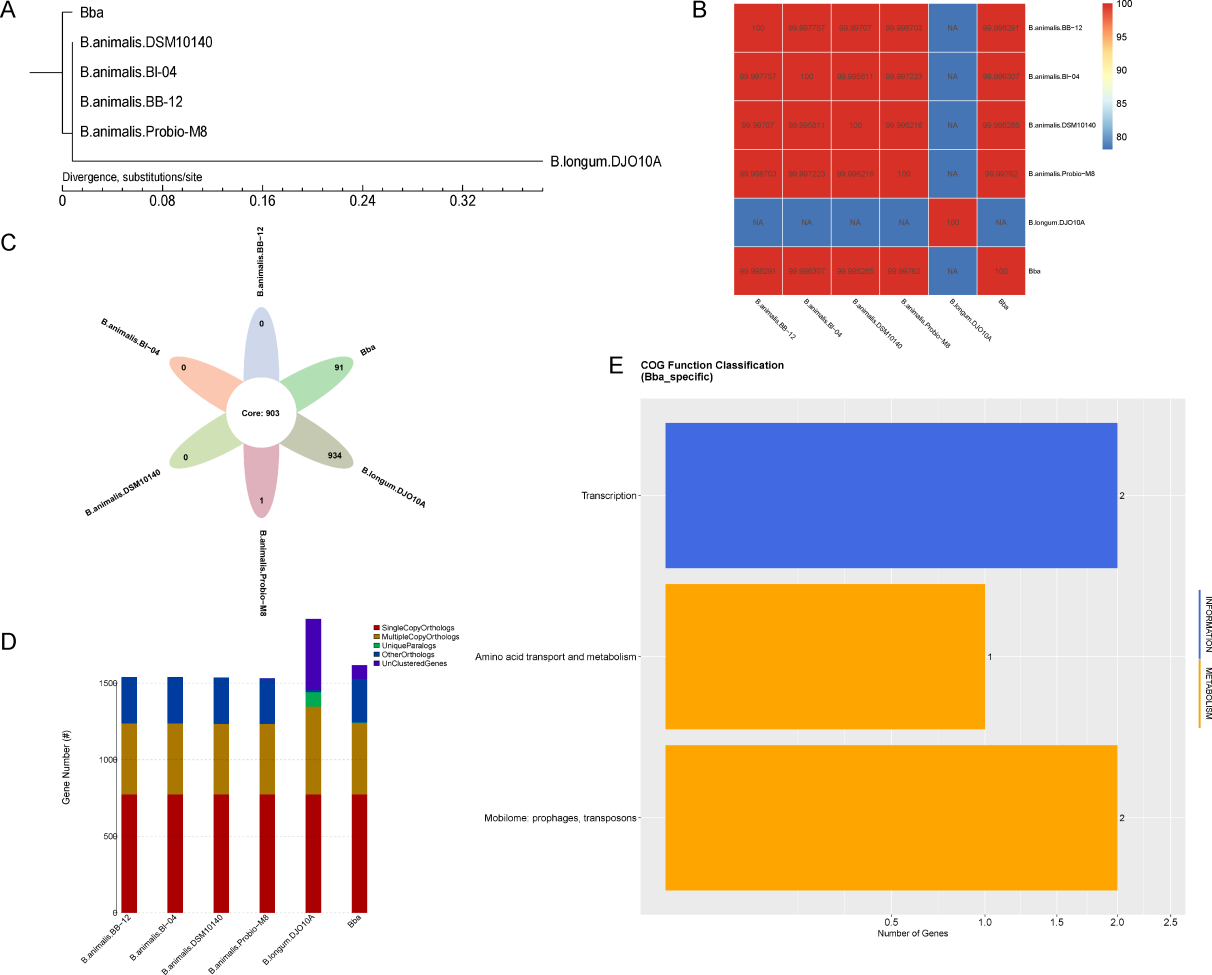
Comparative analysis of six *Bifidobacterium* species (Bba, *B. animalis* DSM10140, *B. animalis* BI-04, *B. animalis* BB-12, *B. animalis* Probio-M8, and *B. longum* DJO10A). The phylogenetic tree is based on core-pan genes (A). Average nucleotide identity (ANI) analysis (B). Venn graph of orthologs in different species gene family (each ellipse represents one strain, and the number in the ellipse means the family number in this species) (C). The classification statistics of genes in each stains (D). COG function classification of *B. animalis* specific genes (E).

For comparative analysis with other *Bifidobacterium* probiotics, *B. animalis* DSM10140, *B. animalis* BI-04, *B. animalis* BB-12, and *B. animalis* Probio-M8 were included, using *Bifidobacterium longum* DJO10A as phylogenetic outgroup (Fig. 4A). According to the average nucleotide identity heatmap, *B. animalis* genome shared similarity over 99.99% with DSM10140, BI-04, Probio-M8, and BB-12 (Fig. 4B). However, besides the common shared genes, *B. animalis* possesses some specific genes, including 89 unclustered genes and 2 unique family (Fig. 4C and D). Compared with DSM10140, BI-04, Probio-M8, and BB-12, *B. animalis* has three specific COG function classifications (transcription, amino acid transport, and metabolism, Mobilome: prophages, transposons) (Fig. 4E). The structural variation (Synteny) at the amino acid level and nucleotide level also demonstrated that *B. animalis* only showed limited genomic structural differences compared with DSM10140, BI-04, Probio-M8, and BB-12 (Fig. S1), indicating close evolutionary distance and probiotic potential.

### Metabolic network reconstruction using genomes of *F. nucleatum* and *B. animalis*

**Fig 5:**
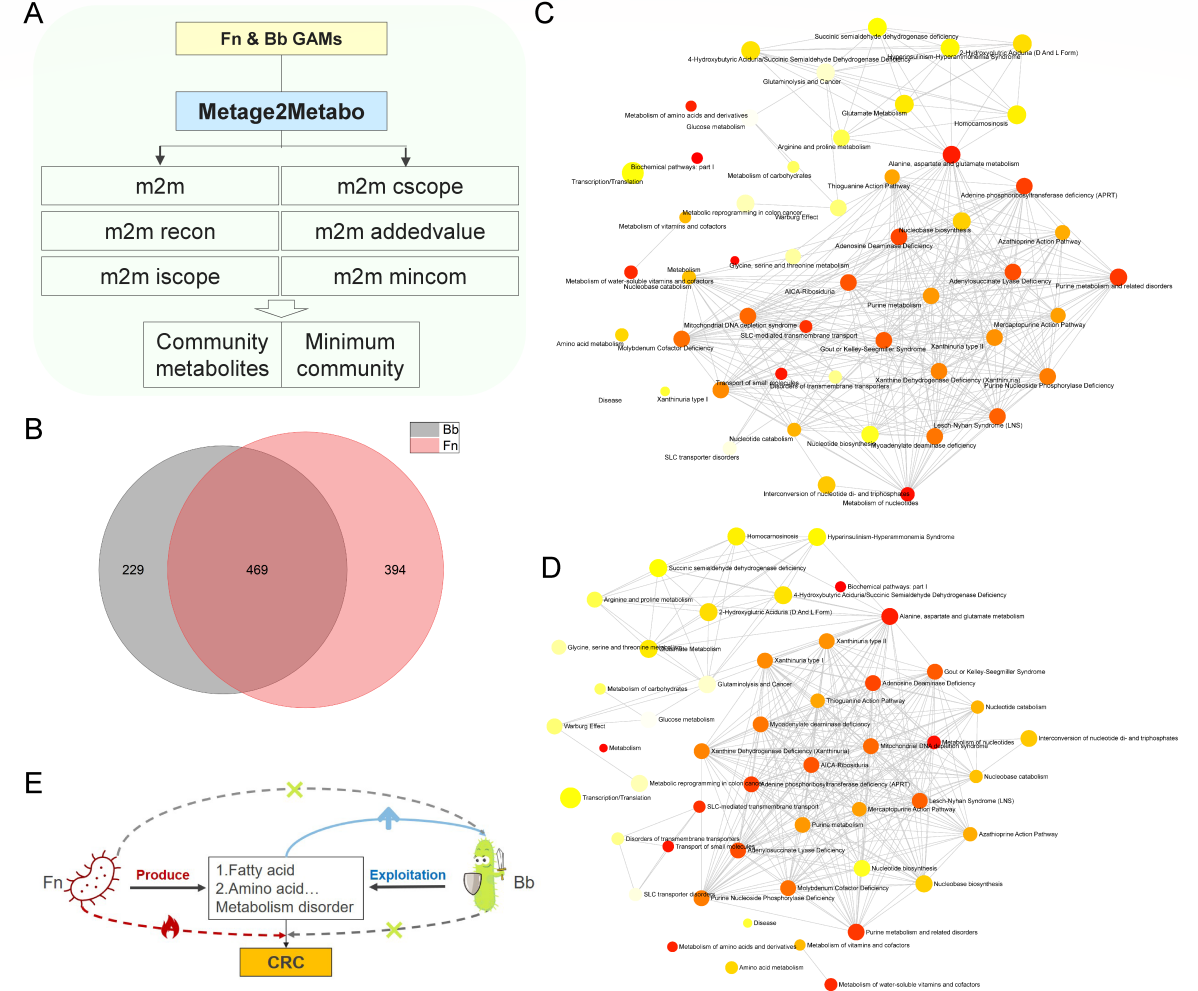
Metabolic network reconstruction and enrichment analysis. Metabolic network reconstruction workflow using Metage2Metabo and Pathway Tool (**A**). Metabolites belong to *B. animalis* and *F. nucleatum* (**B**). Enrichment analysis of *F. nucleatum* specific metabolites (**C**). Enrichment analysis of *B. animalis* specific metabolites (**D**). Potential metabolic interaction mechanism between *B. animalis* and *F. nucleatum* (**E**).

After obtaining the genome information of *B. animalis*, the genome of *F. nucleatum* was downloaded for metabolic network reconstruction together with *B. animalis*. Ninety-seven kinds of nutrients were used as seed files. Following the Metage2Metabo workflow based on the pathway tool, 1,092 metabolites were obtained (Fig. 5A). A total of 229 metabolites were produced only by *B. animalis*, and 394 metabolites were produced only by *F. nucleatum*. A total of 469 metabolites could be produced by both of them (Fig. 5B). Then their unique metabolites were used for enrichment analysis using the MetaboAnalyst database. These *F. nucleatum*-specific metabolites were enriched in alanine, aspartate, and glutamate metabolism, metabolism of nucleotides, Warburg effect, metabolic reprogramming in colon cancer, glycine, serine and threonine metabolism, glutaminolysis, and cancer, etc. (Fig. 5C). Those *B. animalis* specific metabolites were correlated with alanine, aspartate and glutamate metabolism, arginine and proline metabolism, nucleobase biosynthesis, metabolism of carbohydrates, etc. (Fig. 5D). These results demonstrated that competition of the same nutrients was related with similar pathways, yet different metabolites were produced. Therefore, the competition and metabolic inhibition shaped the exploitation relationship (Fig. 5E).

### Metabolic interaction between *F. nucleatum* and *B. animalis*

**Fig 6:**
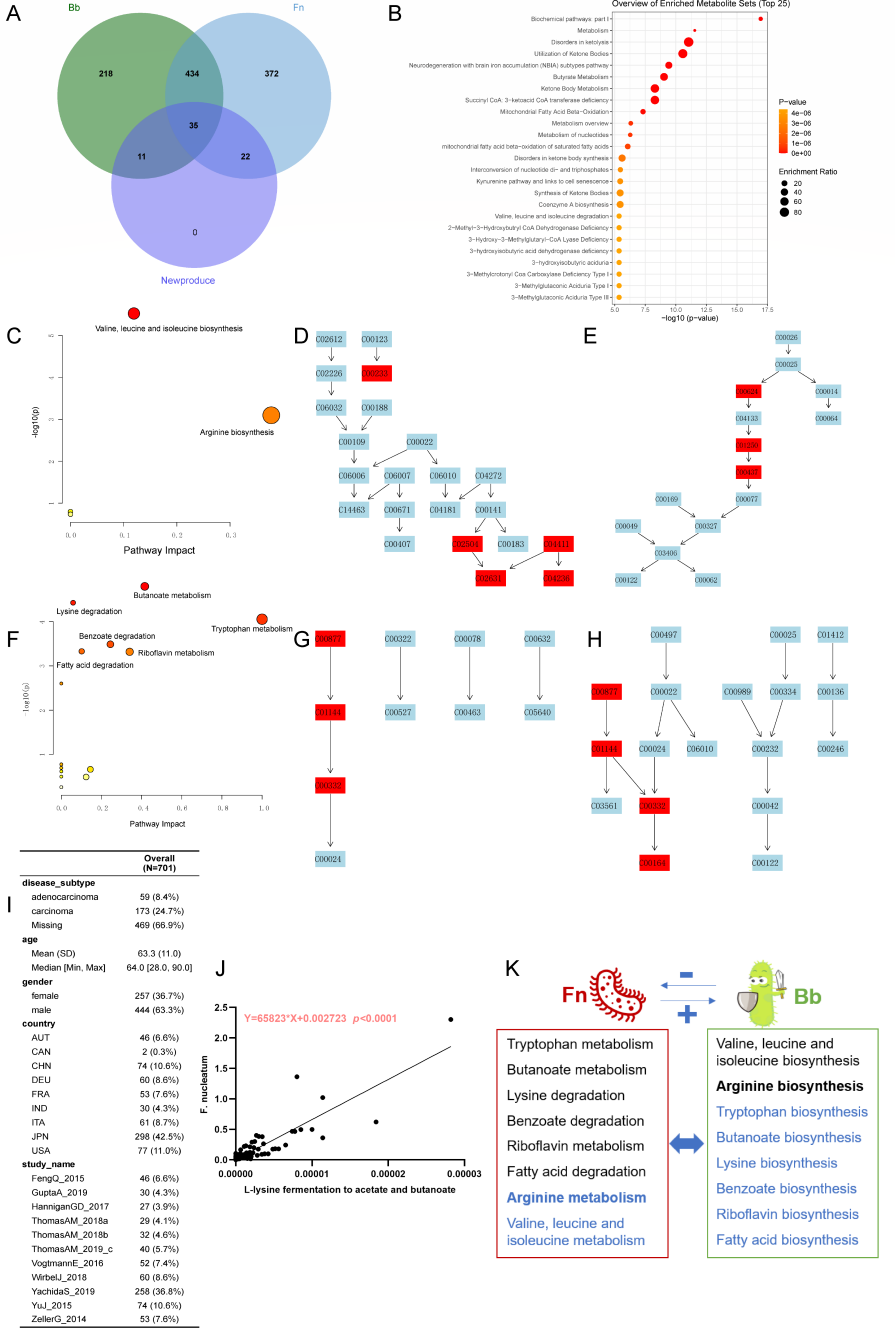
Metabolic interaction between *F. nucleatum* and *B. animalis*. New produced metabolites when both *F. nucleatum* and *B. animalis* exist (**A**). Enrichment analysis of 68 newly produced metabolites (**B**). Targeted pathways annotation of *B. animalis* specific metabolites (**C**). Metabolites in valine, leucine, and isoleucine biosynthesis pathway (**D**). Metabolites in arginine biosynthesis pathway (**E**). Targeted pathways annotation of *F. nucleatum* specific metabolites (**F**). Metabolites in tryptophan metabolism pathway (**G**). Metabolites in butanoate metabolism pathway (**H**). Statistics of CRC fecal samples used for shotgun sequencing (**I**). The relationship between *F. nucleatum* abundance and L-lysine fermentation to acetate and butanoate pathway genes (**J**). The framework of metabolic interaction between *F. nucleatum* and *B. animalis* (**K**).

Among these metabolites, 68 were produced when both *F. nucleatum* and *B. animalis* existed (Fig. 6A; Table S3), which suggested metabolic interaction (cross-feeding) between them. These metabolites were enriched in disorders in ketolysis, utilization of ketone bodies, neurodegeneration with brain iron accumulation subtypes pathway, butyrate metabolism, ketone body metabolism, succinyl CoA: 3-ketoacid CoA transferase deficiency, mitochondrial fatty acid beta-oxidation (Fig. 6B). Then targeted pathway analysis based on model microorganisms were performed. The 11 metabolites (Bb) are mainly related to valine, leucine, and isoleucine biosynthesis and arginine biosynthesis (Fig. 6C through E). The 22 metabolites (Fn) are mainly related to butanoate metabolism, lysine degradation, benzoate degradation, riboflavin metabolism, tryptophan metabolism, fatty acid degradation, etc. (Fig. 6F through H; Fig. S2). Furthermore, we analyzed the shotgun sequencing and function annotation from fecal samples of 701 CRC patients (Fig. 6I). The result also demonstrated that L-lysine fermentation to acetate and butanoate-related genes were positively related with *F. nucleatum* abundance (Fig. 6J). Hence, these new metabolites were considered to satisfy the needs of each other. For instance, arginine biosynthesis belonging to *B. animalis* could meet the needs of *F. nucleatum*. Similarly, the butanoate metabolism in *F. nucleatum* indicated that butanoate produced by *B. animalis* could be used by *F. nucleatum* (Fig. 6K). Therefore, although the metabolic network reconstruction cannot predict growth promotion or inhibition, it can shed light on the metabolic interactions in a community.

### Arginine supplement enhances the competitive ability of *F. nucleatum* against *B. animalis*

Based on the metabolic interaction between *F. nucleatum* and *B. animalis*, the newly produced metabolites may have an influence on the growth of bacteria. *F. nucleatum* is sensitive to acid (34). According to the KEGG annotations, amino acid metabolism and carbohydrate metabolism are the most enriched functions. Thus, we speculated that SCFA produced by *B. animalis*, such as acetic acid and butyric acid, could suppress *F. nucleatum*, while arginine needed by *F. nucleatum* could promote its growth. To verify this hypothesis, qPCR was used to monitor the abundance changes of *F. nucleatum* and *B. animalis*.

**Fig 7:**
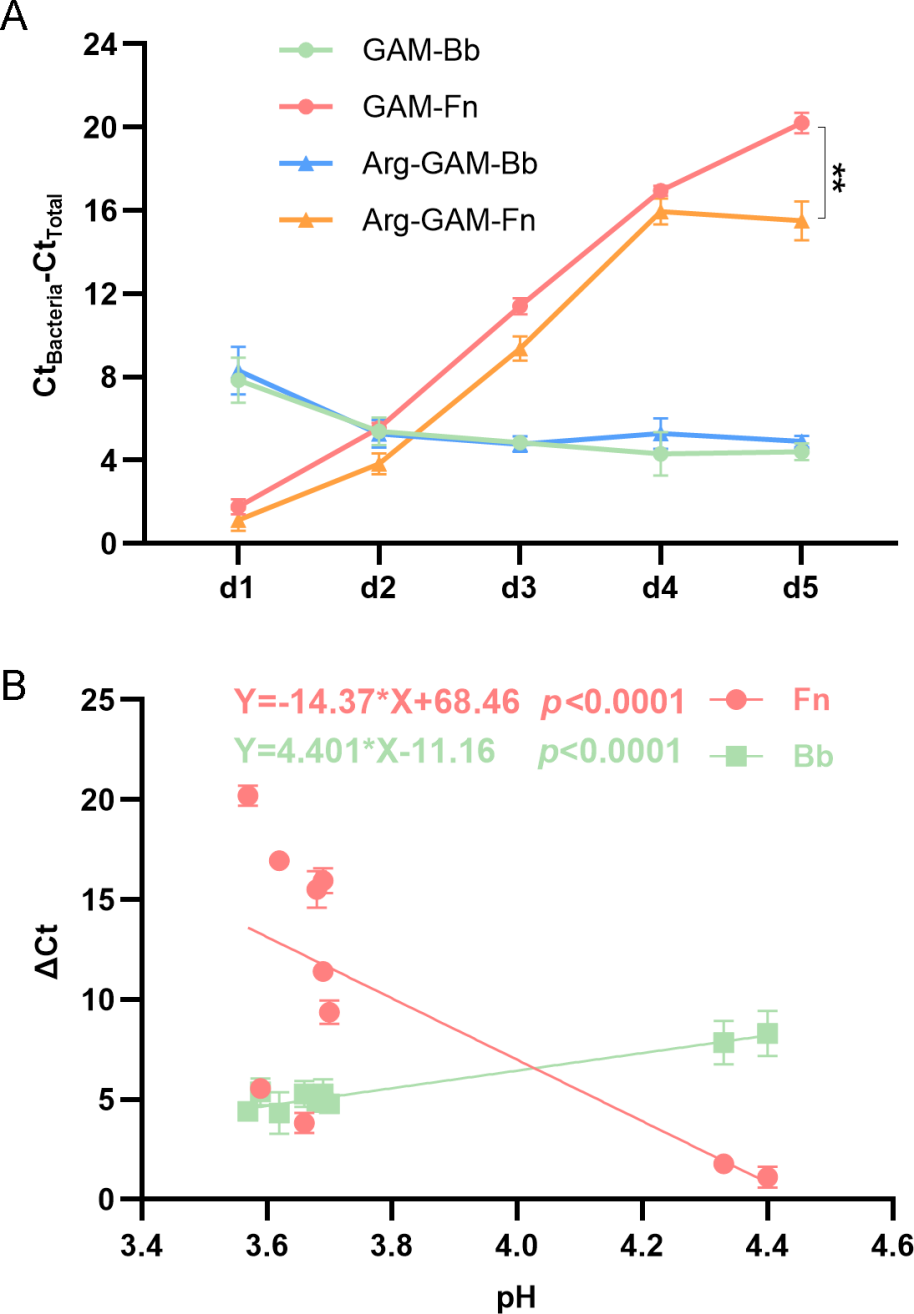
Arginine (Arg) supplement enhances the competitive ability of *F. nucleatum* against *B. animalis*. Growth curve of *F. nucleatum* and *B. animalis* co-cultured in GAM and Arg-GAM medium (**A**). The linear fitting curves of pH values with *F. nucleatum* and *B. animalis*, respectively (**B**).

As shown in Fig. 7A, when they were co-clutured, *F*. *nucleatum* gradually decreased from day 1 to day 5, while *B. animalis* increased and reached stable on the third day. Moreover, the abundance of *F. nucleatum* in Arg-GAM was higher than that in GAM. At the same time, the pH decreased gradually (Fig. S3), and showed a significant negative correlation with *F. nucleatum* abundance and a positive correlation with *B. animalis* (Fig. 7B). However, when arginine was added to the GAM medium, the acid production capacity of *B. animalis* did not show a significant difference. Therefore, the arginine supplement enhanced the competitive ability of *F. nucleatum* against *B. animalis* and resistance against acid, which was consistent with metabolic network reconstruction prediction.

### Arginine metabolism and *Fusobacterium* are enriched in CRC and positively correlated

**Fig 8:**
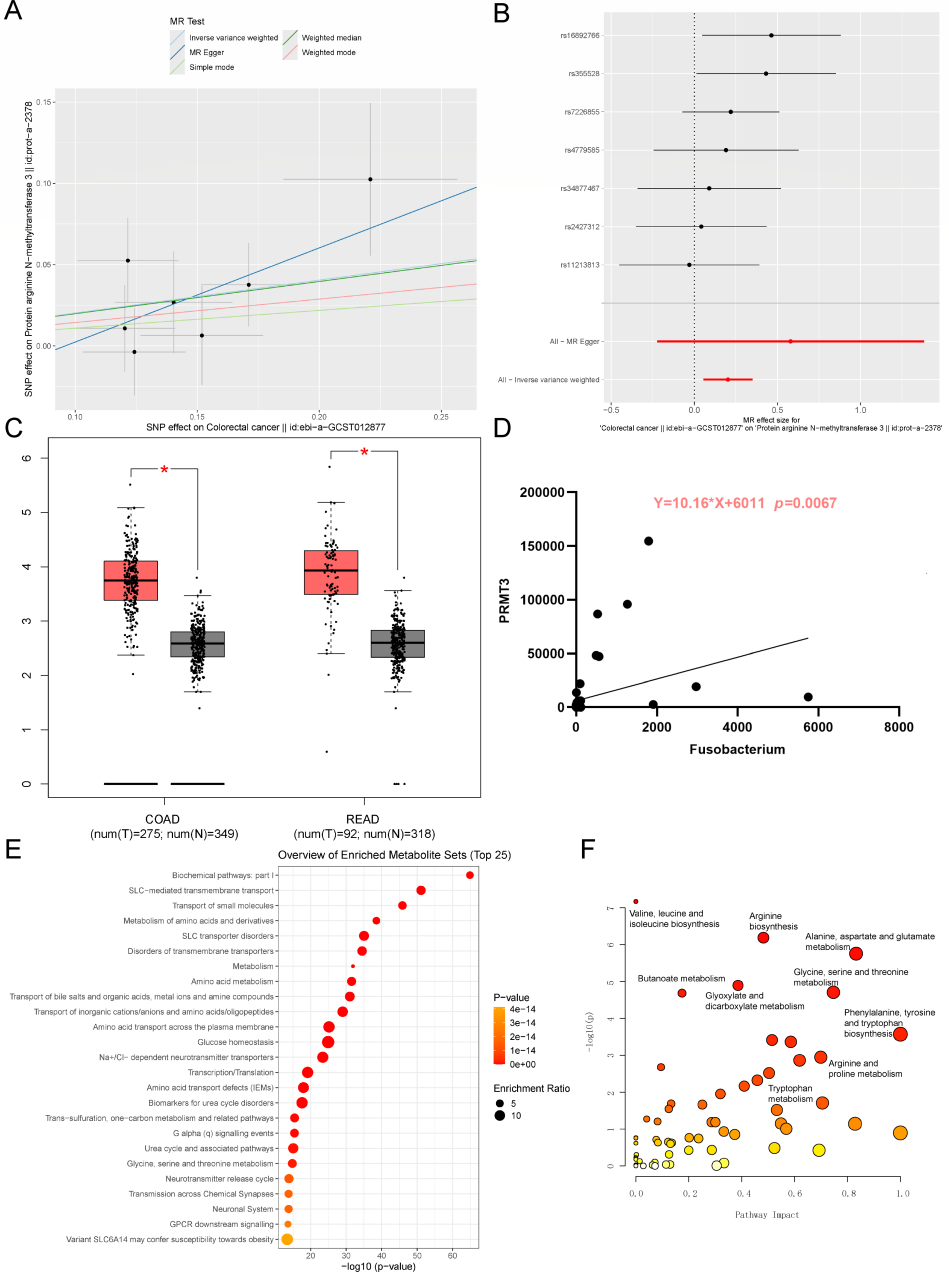
Arginine metabolism is enriched in CRC patients and positively related with *Fusobacterium*. Mendelian randomization of protein arginine N-methyltransferase 3 (PRMT3) and CRC (**A**). Forest analysis of PRMT3 and CRC (**B**). *PRMT3* gene expression in CRC tumors and normal control (**C**). The relationship between *PRMT3* gene expression and *Fusobacterium* abundance in CRC (**D**). Enrichment (**E**) and pathway analysis (**F**) of upregulated metabolites in CRC patients.

To further verify the relationship of arginine metabolism-related genes, CRC and *Fusobacterium*, mendelian randomization analysis, whole-genome sequencing, and transcriptome sequencing of human tissue samples were analyzed. The inverse variance weighted test showed that protein arginine N-methyltransferase 3 (PRMT3) was positively related to CRC (b = 0.2024, *P* = 0.0071), and rs16892766 and rs355528 were the key single nucleotide polymorphisms (SNPs) (Fig. 8A and B). Moreover, PRMT3 is significantly upregulated in CRC tumors (colon adenocarcinoma, COAD; rectum adenocarcinoma, READ) compared with normal control (Fig. 8C). Next, the microbiome in CRC tissues were extracted from TCMA database (The Cancer Microbiome Atlas), which used whole-genome sequencing (species mitigated batch effects were removed and validated by original matched TCGA samples) to obtain tissue-resident microbial profiles (35). Then the gene expression RNAseq data were obtained from the UCSC Xena database (36). After selecting the matched samples, 62 CRC samples were used for further analysis and the *Fusobacterium* abundance was significantly related to PRMT3 expression (Fig. 8D). Arginine methylation is a post-translational modification process, which is catalyzed by PRMT. PRMT3 plays a key role in the tumorigenesis process and serves as a potential therapeutic target (37). Finally, metabolome analysis from MACdb showed that the metabolism of amino acids and derivatives was significantly upregulated in CRC patients compared to healthy control (Fig. 8E) (38). Especially, valine, leucine, and isoleucine biosynthesis, arginine biosynthesis, and alanine, aspartate, and glutamate metabolism are the top three enriched pathways (Fig. 8F). Therefore, a diet rich in arginine may promote the growth of *F. nucleatum* and exacerbate CRC, while *B. animalis* is a potential probiotic to treat *F. nucleatum*-infected CRC.

## DISCUSSION

In this study, the abundance changes of CRC-related harmful and beneficial bacteria was analyzed. Especially, *F. nucleatum* was enriched in CRC patients while *B. animalis* decreased. *In vitro* experiments showed that *B. animalis* could inhibit *F. nucleatum* in exploitation manner. Whole-genome sequencing and metabolic network reconstruction revealed that nutritional competition and acidic molecules produced by *B. animalis* via carbohydrate metabolism played a key role. The arginine supplement could rescue *F. nucleatum* growth, collaborating with the model prediction.

Eliminating *F. nucleatum* was beneficial for CRC patients, and microbial-based therapy is promising. Since 2013, fecal microbiota transplantation has been approved by the FDA and achieved great success in *Clostridium difficile* infection treatment (39). However, targeted clearance of *F. nucleatum* strategies is still limited. Although *Akkermansia muciniphila* and *Saccharomyces cerevisiae* JKSP39 showed ameliorative effects on *F. nucleatum*-related periodontitis and colitis, the detailed mechanism is deficient, which is essential for further clinical translation. Probiotic actions of bifidobacteria are often with strain-specificity (30). In this study, *B. animalis* was proposed as a potential probiotic for its inhibition on *F. nucleatum* partly based on acid metabolites. Usually, lactic acid, acetic acid, propionic acid, and butyric acid are the common acidic small molecules produced by *Bifidobacterium*. Moreover, the exploitation relationship between them indicates that cross-feeding exists. Using traditional molecular biology-related methods to elucidate mechanisms is complicated and laborious (32). Here, we explored this issue from the perspective of genome and metabolic networks.

Through whole-genome sequencing, the genomic characteristics and genes related to probiotic properties can be obtained (40). In the KEGG function annotations of *B. animalis* genome, most genes belonged to amino acid metabolism and carbohydrate metabolism pathways. Enzymes such as beta-glucosidase, beta-glucosidase, isoamylase, xylose isomerase, and acetate kinase, were identified, and these carbohydrate metabolism-related enzymes will produce abundant acidic metabolites to lower pH. Additionally, the bile salt hydrolase gene is also identified, and it can hydrolyze conjugated bile acid to release free bile acid, which was reported to have antibacterial activity (41). Therefore, we speculate that *B. animalis* may generate antibacterial activity in various ways *in vivo*.

Besides genome sequencing, the metabolome is a more intuitive reflection of metabolic activity. Previous studies used non-targeted and targeted metabolomes to reveal the active metabolites for CRC suppression (42, 43). By metabolic simulation based on bacterial genome, the efficiency has been greatly improved. Over the last decades, various genome-scale metabolic networks were developed, such as Pathway Tools (44), CarveMe (45), KBase (46), and MiSCoTo (45). Metage2Metabo based on Pathway Tools was selected for its advantages in the identification of critical species with respect to metabolic cooperation, accession of the cooperation potential between species and characterization of individual metabolisms and collective metabolic complementarity (47). According to the analysis results, the individual metabolisms and collective metabolisms were successfully obtained. For *F. nucleatum*, leucine and isoleucine biosynthesis and arginine biosynthesis in *B. animalis* may reflect its metabolic demand. The major resources of arginine are arginine-enriched nutrition supplements from dietary intake, endogenous synthesis from citrulline, and protein catabolism. It can be metabolized into NO and citrulline by nitric oxide synthase, ornithine and urea by arginase, and agmatine by arginine decarboxylase (48), which then promotes the development of CRC. It was reported that arginine methylation is a common post-translational modification, being carried out by the nine members of the PRMT family (49). PRMTs tend to be upregulated in many cancers and arginine methylation deregulation was reported in numerous reports, such as leukemia and lymphoma, brain cancer, lung cancer, breast cancer, and CRC (50). Especially, PRMT1 expression was elevated in CRC cell lines and tissues and promoted glycolysis, proliferation, and tumorigenesis by phosphoglycerate kinase 1 mediation (51). Our result showed that the *Fusobacterium* abundance was significantly related to PRMT3 expression. Arginine supplement promoted the growth of *F. nucleatum* when co-cultured with *B. animalis*. Recent studies demonstrated that PRMT3 was upregulated in CRC and related to poor overall survival. It stabilized HIF1α by modulating the HIF1/VEGFA signaling pathway and promoted C-MYC stabilization, thus playing oncogene functions (52, 53). However, the relationship between *F. nucleatum* and PRMT3 deserves further elucidation. Recently, some studies revealed that *F. nucleatum* infection increased METTL3-mediated m6A methylation to promote CRC proliferation (54). Both METTL3 and METTL4 are common m6A regulators, and PRMT3-mediated arginine methylation of METTL14 can promote malignant progression (55, 56). Therefore, *F. nucleatum* infection may promote PRMT3 upregulation and METTL3-mediated m6A methylation to induce carcinogenesis, which needs verification in the future. Consequently, genome-scale metabolic network reconstruction provides valuable information about complex metabolic systems.

There are several limitations in this study. First, the genome-scale metabolic network reconstruction cannot accurately predict the influence of metabolites on bacteria themselves, and the promoting or inhibiting effects rely on prior knowledge, then further experimental verification is needed. Second, the metabolic dynamics are affected by the external environment, such as nutrients and gut microbiota, then the antibacterial activity *in vivo* requires further exploration. Finally, the safety and effectiveness of *B. animalis* need to be confirmed through clinical trials.

In summary, our study confirmed the *F. nucleatum* enrichment and *B. animalis* depletion in CRC patients compared with healthy control. As a potential probiotic, *B. animalis* could inhibit *F. nucleatum* growth. Whole-genome sequencing demonstrated its carbohydrate metabolism function and close phylogenetic relationship with known probiotics. Metabolic network reconstruction revealed metabolic interaction between them, which was verified by the arginine supplement experiment and mendelian randomization analysis, thus highlighting the potential of genome sequencing and genome metabolic network reconstruction for probiotic function mining.

## MATERIALS AND METHODS

### Culture of *F. nucleatum* and *B. animalis*

*F. nucleatum* (ATCC 25586) is cultured in Gifu Anaerobic Medium (GAM, 0.1% VK_1_ and 5 mg/mL Hemin) and *B. animalis* (CGMCC 1.15623, isolated from infant feces) is cultured in MRS medium. They were cultured in anaerobic conditions (5% CO_2_, 10% H_2_, 85% N_2_) at 37°C. The growth curve was recorded at an absorbance of 600 nm.

### Plate growth inhibition assay

*F. nucleatum* was diluted to 10^7^ CFU/mL and spread on a GAM plate, containing wells with a diameter of 6 mm, then 140 µL *B. animalis* (10^8^ CFU/mL) was added into the well. After 48 h of cultivation, the zone of inhibition was recorded.

For the competition assay, 10 µL *F*. *nucleatum* and 10 µL *B. animalis* (10^8^ CFU/mL) were spread on a GAM plate and adjacent to each other, then it was cultured for 48 h.

### Genomic extraction, PCR amplification, and 16S rRNA sequencing

*F. nucleatum* and *B. animalis* at logarithmic growth period were used for genomic extraction. 1 mL bacterial fluid was centrifuged at 8,000 r/min for 5 min, and the genome was extracted using the TIANamp Bacteria DNA Kit following the manufacturer’s instructions (TIANGEN Biotech Co., Ltd.). The PCR (95°C, 5 min, 30 cycles for 95°C 30 s, 56°C 30 s, 72°C 90 s, finally 72°C 10 min) were performed with 27F： TACGGYTACCTTGTTACGACTT and 1492R: AGAGTTTGATCMTGGCTCAG. Then the products were subjected to Sanger sequencing (Sangon Biotech Co., Ltd.) and BLAST in NCBI for identification.

### Phylogenetic tree construction

16S RNA sequences were downloaded from NCBI and used for multiple sequence alignment. Next, the aligned result was used for phylogenetic tree construction based on the Neighbor-joining method (Bootstrap Replications, 500; Model, Maximum Composite Likelihood; Rates among Sites, Uniform Rates; Gaps/Missing Data Treatment, Pairwise deletion; Substitutions to Include, d: Transitions + Transversions). All the steps were performed using MEGA X (57).

### Meta statistics of *F. nucleatum* distribution globally

The keywords “*F. nucleatum* and CRC,” “*F. nucleatum* and colon cancer,” “*F. nucleatum* and rectum cancer,” and “*F. nucleatum* and cancer” were used for search in PubMed and Google Scholar. Review and non-English literature were excluded. The sample types and country of origin were extracted. The information was summarized in Excel and visualized with R software.

### Whole-genome sequencing of *B. animalis* and genome assembly

*B. animalis* was cultured in MRS medium for 24 h, then centrifuged at 8,000 r/min for 10 min. Bacterial precipitation was collected and used for whole-genome sequencing (BGI). The sample integrity and purity were detected by agarose gel electrophoresis (1%). 1 µg genomic DNA was randomly fragmented by Covaris and was selected by Agencourt AMPure XP-Medium kit to an average size of 200–400 bp. After the end-repaired and then 3′ adenylated, adaptors were ligated to these 3′ adenylated fragments. Then PCR products were purified, heat denatured, and circularized. Single-strand circle DNA (ssCir DNA) was formatted as the final library and was sequenced by BGISEQ-500. After filtering the raw data, K-mer analysis was used to estimate the size of the genome, degree of heterozygosis, and degree of duplication. Based on the valid data, the optimal assembly results were obtained after multiple adjustments.

### Bioinformatics analysis of *B. animalis* genome

Glimmer software was used to predict genes of assembly. RNAmmer, tRNAscan, and Infernal were used to predict rRNAs, tRNA, and sRNAs, respectively (58, 59). Next, the Tandem Repeat Finder was used to predict tandem repeat sequence (TR), minisatellite, and minisatellite sequences. Using PhiSpy and CRISPRCasFinder to identify prophages and CRISPRs (60–62).

The function annotation was accomplished by analysis of protein sequences, which were obtained by aligning genes with databases using Diamond, including GO, KEGG, COGs of proteins, Swiss-Prot, NR, EggNOG, Antibiotic Resistance Genes Database (ARDB), Comprehensive Antibiotic Resistance Database, Fungal Cytochrome P450 Database, Carbohydrate-Active enZYmes Database (CAZy), virulence factor database (VFDB), Type III secretion system Effector protein (T3SS), TransportDB, and Antibiotic Resistance Gene-ANNOTation (ARG-ANNOT) databases (63, 64).

### Genome-scale metabolic network reconstruction

The genomes of *B. animalis* and *F. nucleatum* were used for metabolic network reconstruction, which was achieved with an automated command-line version of PathwayTools (65). Then the whole process was finished using the Metage2Metabo (M2M) tool (47).

First, annotated genomes were used to reconstruct draft metabolic networks with the m2m recon commands. In this process, seeds were used, which were a set of compounds that define the nutritional conditions of the community in SBML format (47). Additionally, to model the environment *in vivo*, glycochenodeoxycholic acid and glycocholic acid (from the MetaCyc database) were added to the seed. Then command iscope and cscope were used to predict individual metabolic potentials and metabolic potential of the community, respectively. Next, m2m added value was used to compute the added value of combining metabolisms in the microbiota (such as metabolic cooperation) with respect to studying individually the metabolism of each organism.

Based on the metabolic network, the selected metabolites were subjected to enrichment analysis and targeted pathway analysis using MetaboAnalyst 5.0 (66). For *B. animalis* pathway analysis, *Bifidobacterium longum* NCC2705 was used as the model strain. Similarly, *Escherichia coli* K-12 MG1655 was used for *F. nucleatum* metabolite pathway annotation.

### Real-time quantitative PCR assay

*F. nucleatum* and *B. animalis* were inoculated in GAM and Arg-GAM (0.1% arginine). After 24 h cultivation, the bacteria were adjusted to OD_600nm_ = 1.0, and 0.4 mL was inoculated in new GAM and Arg-GAM. The mixed bacterial solution was cultivated for five generations. For each generation, the genome was extracted using the TIANamp Bacteria DNA Kit. Primers (Fn-F: 5′-CAACCATTACTTTAACTCTACCATGTTCA-3′, Fn-R: 5′-GTTGACTTTACAGAAGGAGATTATGTAAAAATC-3′. Bb-F: 5′-CATCGCTTAACGGTGGAT-3′, Bb-R: 5′-TTCGCCATTGGTGTTCTT-3′. 63F: 5′-GCAGGCCTAACACATGCAAGTC-3′, 335R: 5′-CTGCTGCCTCCCGTAGGAGT-3′) were used to monitor the abundance of *F. nucleatum* and *B. animalis*. 63F and 335R were used as internal references.

qPCR was performed following the manufacturer’s instructions (servicebio, Wuhan). The 10 µL reaction mix included 5 µL 2× Mix (low ROX), 0.3 µL forward primer, 0.3 µL reverse primer, 1 µL DNA template, and 3.4 µL ddH_2_O. The reaction procedure was 95°C 30 s, 40 cycles of 95°C 15 s, 60°C 60 s. Then a melting curve procedure was added to verify the primer specificity.

### Mendelian randomization analysis

The Genome-wide association studies (GWAS) data of CRC (ebi−a−GCST012877) and Protein arginine N-methyltransferase 3 (prot−a−2378) were obtained from IEU OpenGWAS database, and SNPs associated with exposure were extracted at the genome-wide significance level (threshold alpha = 5 × 10^−8^). Then, r2 < 0.01 and kb = 10,000 were used to remove SNPs with chained disequilibrium. MR Egger regression, Weighted median, Inverse variance weighted, Simple mode, and Weighted mode were used to test the importance of SNPs. Q-test was performed to evaluate the heterogeneity. Statistical power and leave-one-out sensitivity analyses were conducted with individual SNPs. All the analyses were finished using the TwoSampleMR package.

### Statistical analysis

All statistical analyses were performed using R V.3.6.0 and GraphPad Prism. Differences between groups were obtained by Kruskal-Wallis or t-tests, and a *P* value < 0.05 was considered statistically significant.

## Data Availability

All clean sequence data for this work have been deposited to the NCBI database (PRJNA1182847, CP173197.1).

## SUPPLEMENTAL MATERIAL

Supplemental material spectrum.02235-24-s0001.docx Tables S1 to S3; Fig. S1 to S3.

ASM does not own the copyrights to Supplemental Material that may be linked to, or accessed through, an article. The authors have granted ASM a non-exclusive, world-wide license to publish the Supplemental Material files. Please contact the corresponding author directly for reuse.
