# Supplementary material for: Interspecies interactions mediated by arginine metabolism enhance the stress tolerance of Fusobacterium nucleatum against Bifidobacterium animalis
Source: Microbiol Spectr. 2025 Jan 27;13(3):e02235-24. doi: 10.1128/spectrum.02235-24 (PMC11878013; doi:10.1128/spectrum.02235-24)

**Supplementary Tables**

**Table Sl Basic information of *Bifidobacterium animalis* genome.**

| **Type** | **Seq Length (bp)** | **Num of gene** | **GC content or**  **Average Length** | **Length / Genome Length (%)** |
| --- | --- | --- | --- | --- |
| chromosome | 1,944,145 | 1,617 | 60.48% | 86.75 |
| tRNA | 4,000 | 53 | 75.47 bp | 0.2057 |
| 5s rRNA | 4 | 468 | 117 bp | 0.0240 |
| 16s rRNA | 4 | 6,104 | 1,526 bp | 0.3139 |
| 23s rRNA | 4 | 12,351 | 3,087.75 bp | 0.6352 |
| sRNA | 1 | 125 | 125 bp | 0.0064 |

**Table S2 Statistical function analysis of *Bifidobacterium animalis* gene.**

| **Name** | **Database** | | | | | | |
| --- | --- | --- | --- | --- | --- | --- | --- |
|  | Total | VFDB | ARDB | CAZY | IPR | SWISSPROT |  |
| Bba | 1,617 | 68 | 2 | 102 | 1,352 | 641 |  |
|  | COG | CARD | GO | KEGG | NR | T3SS |  |
|  | 1,161 | 2 | 1,030 | 1,060 | 1,616 | 349 |  |

**Table S3 New produced metabolites in the community.**

| **New produced metabolites** | **Metabolites** |
| --- | --- |
| Common metabolites | 4'-phosphopantetheine, dCTP, UDP-N-acetyl-alpha-D-glucosamine-enolpyruvate, malonyl-CoA, O-succinyl-L-homoserine, 4-aminobenzoate, N5-carboxyaminoimidazole ribonucleotide, 5-amino-1-(5-phospho-beta-D-ribosyl)imidazole, 2-(formamido)-N1-(5-phospho-beta-D-ribosyl)acetamidine, 5-amino-1-(5-phospho-D-ribosyl)imidazole-4-carboxylate, 5'-phosphoribosyl-4-(N-succinocarboxamide)-5-aminoimidazole, quinolinate, 2-iminosuccinate, palmitoyl-CoA, N2-formyl-N1-(5-phospho-beta-D-ribosyl)glycinamide, succinate, succinyl-CoA, adenine  N-acetyl-alpha-D-glucosamine 1-phosphate, dCMP, dCDP, L-cystathionine, 3'-dephospho-CoA, dGTP, acetyl-CoA, coenzyme A, dUTP, dUMP, L-homocysteine, hydrogen sulfide, O-acetyl-L-serine, UDP-N-acetyl-alpha-D-muramate, UDP-N-acetyl-alpha-D-glucosamine, UDP-N-acetyl-alpha-D-muramoyl-L-alanine, UDP-N-acetyl-alpha-D-muramoyl-L-alanyl-D-glutamate |
| Bb metabolites | 2-isopropylmaleate, (2S)-2-isopropylmalate, (2R,3S)-3-isopropylmalate, (2S)-2-isopropyl-3-oxosuccinate, formyl-CoA, N-acetyl-L-methionine, N-acetyl-L-glutamate, N-acetylglutamyl-phosphate, N-acetyl-L-ornithine, N-acetyl-L-glutamate 5-semialdehyde, 4-methyl-2-oxopentanoate |
| Fn metabolites | N2-succinylglutamate, (3S)-citryl-CoA, 7,8-dihydroneopterin 3'-triphosphate, cyclic-GMP, adenine ribotide phosphate, crotonyl-CoA, (S)-3-aminobutanoyl-CoA, acetoacetate  (S)-3-hydroxybutanoyl-CoA, acetoacetyl-CoA, CoA-disulfide, 5-amino-6-(5-phospho-D-ribitylamino)uracil, 5-amino-6-(5-phospho-D-ribosylamino)uracil, 6,7-dimethyl-8-(1-D-ribityl)lumazine, 5-amino-6-(D-ribitylamino)uracil, 2,5-diamino-6-(5-phospho-D-ribosylamino)pyrimidin-4(3H)-one, UDP-2-acetamido-2,6-dideoxy-beta-L-arabino-hex-4-ulose, 2'-(5''-triphospho-alpha-D-ribosyl)-3'-dephospho-CoA  lauroyl-CoA, 4-amino-4-deoxychorismate, (R)-4'-phosphopantothenoyl-L-cysteine, carbon monoxide |

**Supplementary Figure legends**

**Figure S1.** Structural Variation (Synteny) analysis. *B. animalis* (Bba) and *B. animalis* BB-12 synteny at nucleic acid level (A). Bba and *B. animalis* BB-12 synteny at animo acid level (B). Bba and *B. animalis* Bl-04 synteny at nucleic acid level (C). Bba and *B. animalis* Bl-04 synteny at animo acid level (D). Bba and *B. animalis* DSM10140 synteny at nucleic acid level (E). Bba and *B. animalis* DSM10140 synteny at animo acid level (F). Bba and *B. animalis* Probio-M8 synteny at nucleic acid level (G). Bba and *B. animalis* Probio-M8 synteny at animo acid level (H). Bba and *B. longum* DJO10A synteny at nucleic acid level (I). Bba and *B. longum* DJO10A synteny at animo acid level (J).

**Figure S2.** Targeted pathways analysis of *F. nucleatum* specific metabolites. Lysine degradation (A). Benzoate degradation (B). Riboflavin metabolism (C). Fatty acid degradation (D).

**Figure S3.** pH changes of GAM and Arg-GAM supernatant.

**Figure S1**





**Figure S2**


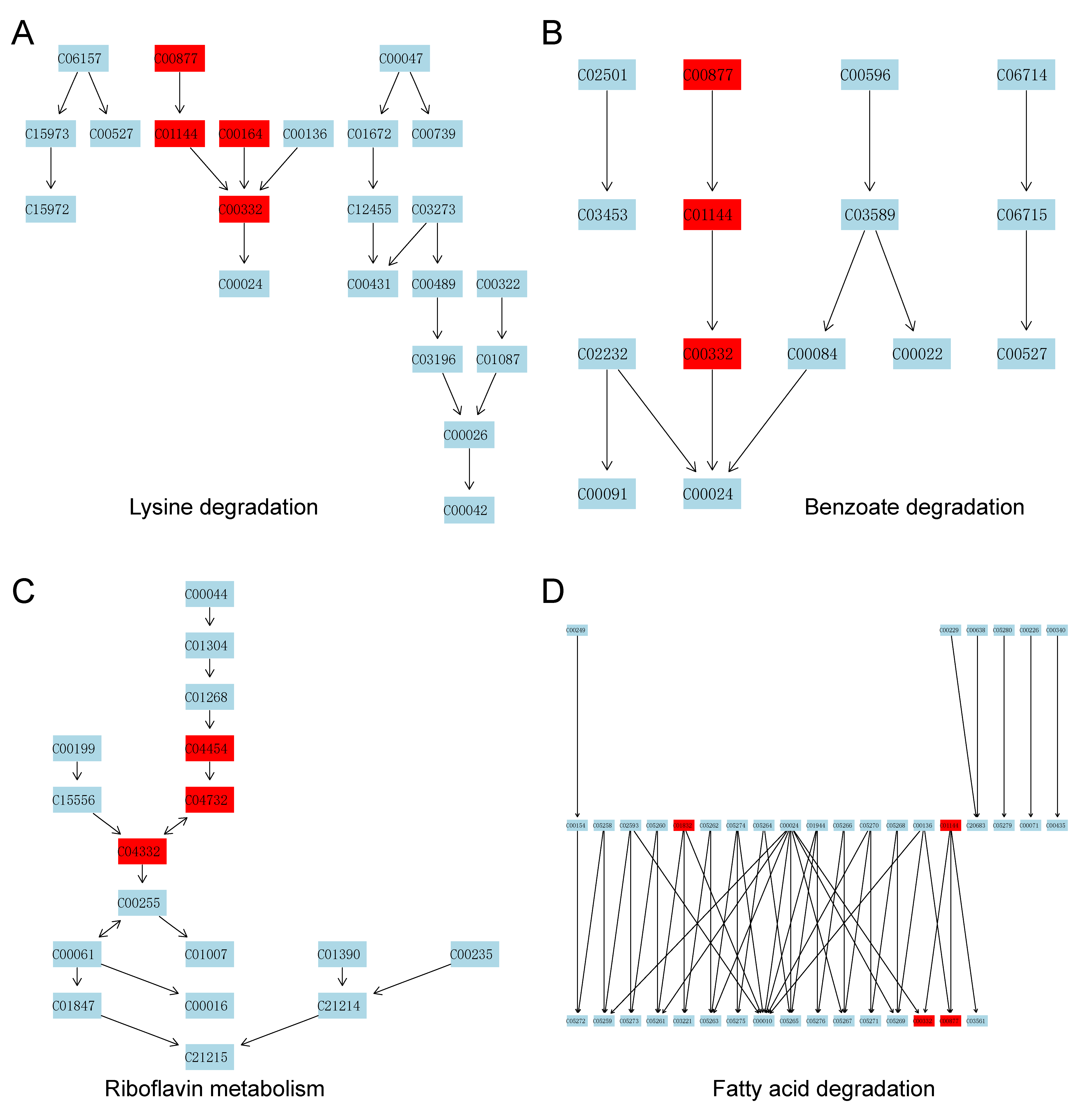


**Figure S3**


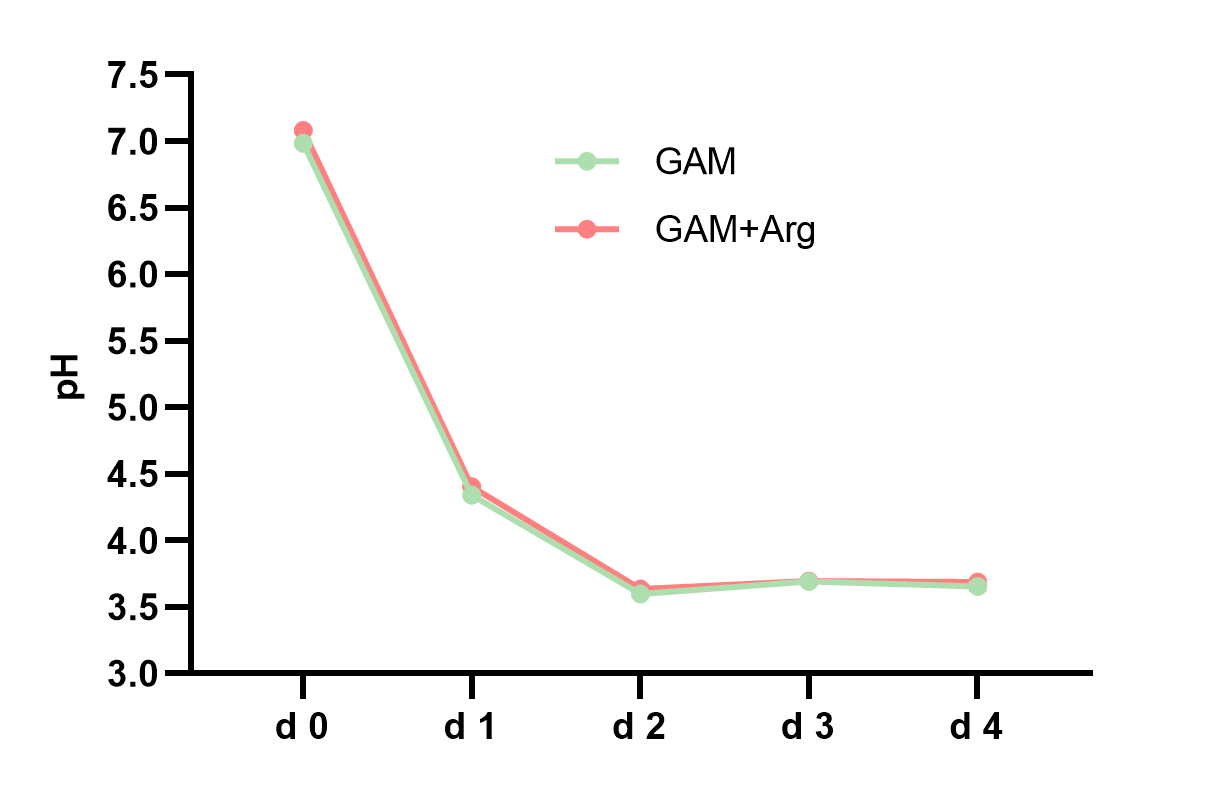

Supplement: Supplemental material — Tables S1 to S3; Fig. S1 to S3. [file spectrum.02235-24-s0001.docx]
